# Elevated Brain Glutamate Levels in Bipolar Disorder and Pyruvate Carboxylase-Mediated Anaplerosis

**DOI:** 10.3389/fpsyt.2021.640977

**Published:** 2021-02-23

**Authors:** Jun Shen, Jyoti Singh Tomar

**Affiliations:** Section on Magnetic Resonance Spectroscopy, Molecular Imaging Branch, National Institute of Mental Health Intramural Research Program, National Institutes of Health, Bethesda, MD, United States

**Keywords:** glutamate, magnetic resonance spectroscopy, pyruvate carboxylase, bipolar disorder, obesity

## Abstract

*In vivo* ^1^H magnetic resonance spectroscopy studies have found elevated brain glutamate or glutamate + glutamine levels in bipolar disorder with surprisingly high reproducibility. We propose that the elevated glutamate levels in bipolar disorder can be explained by increased pyruvate carboxylase-mediated anaplerosis in brain. Multiple independent lines of evidence supporting increased pyruvate carboxylase-mediated anaplerosis as a common mechanism underlying glutamatergic hyperactivity in bipolar disorder and the positive association between bipolar disorder and obesity are also described.

## Introduction

The etiologic and disease mechanisms of bipolar disorder remain poorly understood. A growing body of evidence indicates a central role of mitochondrial dysfunction in the pathophysiology of bipolar disorder. Post-mortem brain studies have revealed abnormal size, structure and distribution of mitochondria as well as a pronounced and extensive decrease in nuclear gene expression governing oxidative phosphorylation in bipolar disorder (1–3). These post-mortem results are consistent with *in vivo* findings of elevated cerebrospinal fluid pyruvate and lactate levels (4, 5), decreased adenosine triphosphate production and a significant shift from oxidative phosphorylation to glycolysis in brain in bipolar disorder accompanied by elevated brain lactate levels and lowered intracellular pH as reported by *in vivo* ^31^P and ^1^H magnetic resonance spectroscopy (MRS) studies (6–10). Paradoxically, despite the impaired mitochondrial function and oxidative metabolism in bipolar disorder *in vivo* ^1^H MRS studies have also reported a highly reproducible pattern of elevated total glutamate or glutamate + glutamine levels (11) (glutamate + glutamine is dominated by glutamate in MRS spectra).

Pyruvate carboxylase is a mitochondrial enzyme. It catalyzes the thermodynamically irreversible carboxylation of pyruvate to oxaloacetate which is a tricarboxylic acid (TCA) cycle intermediate used for various biosynthetic pathways depending on the tissues. The biotin-dependent pyruvate carboxylase employs pyruvate and the polar molecule bicarbonate instead of CO_2_ as its substrates:

$$\begin{array}{l} \text{pyruvate}+{\text{HCO}}_{3}^{-}+\text{adenosine triphosphate }\to \text{oxaloacetate }+\text{adenosine diphosphate}\\ \;+\text{inorganic phosphate} \end{array}$$

**Figure 1:**
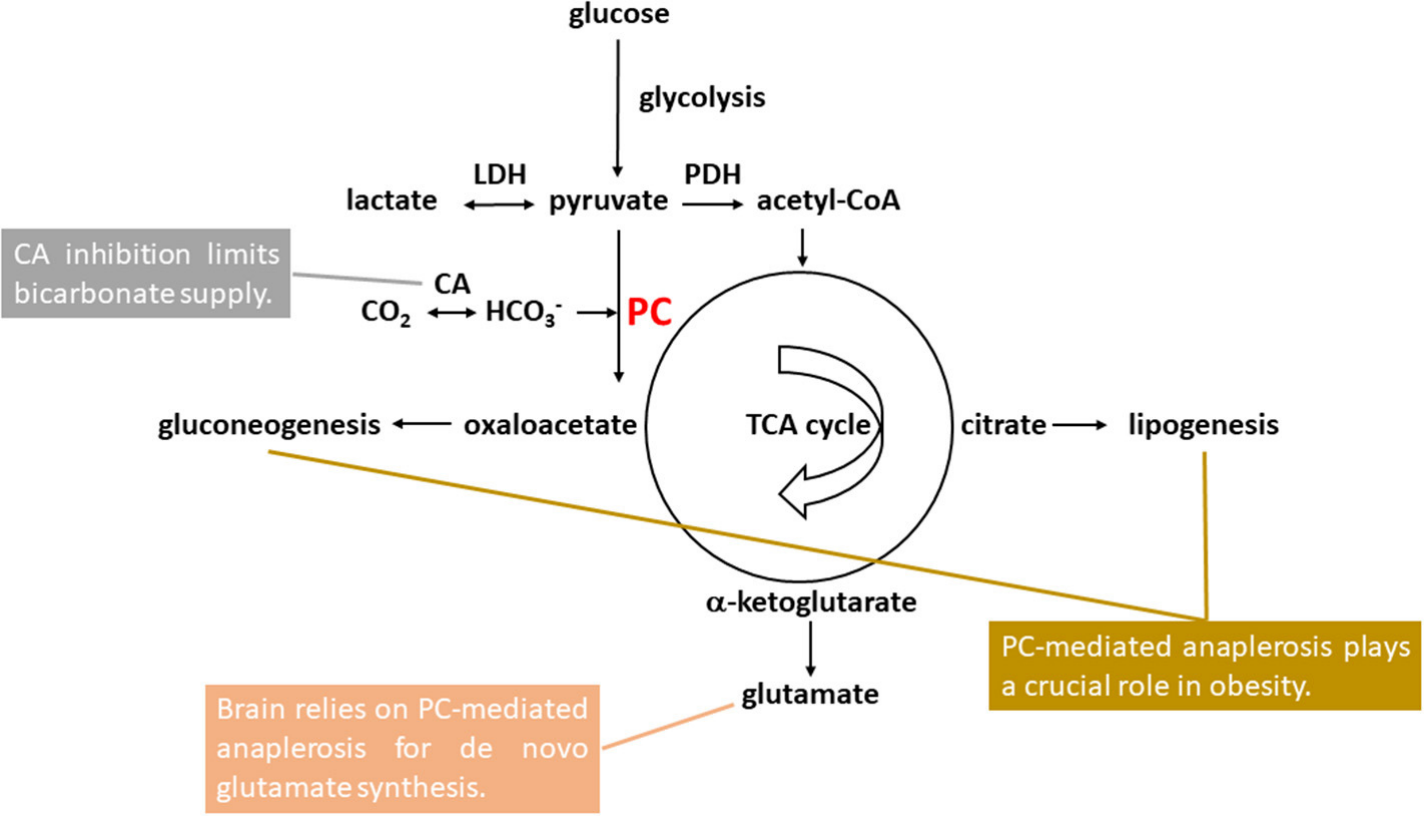
Pyruvate carboxylase (PC)-mediated anaplerosis in bipolar disorder and comorbid obesity. Glucose first undergoes glycolysis with pyruvate as the end product. Pyruvate is in rapid exchange with lactate catalyzed by lactate hydrogenase (LDH). Brain pyruvate and lactate levels were found to be elevated in bipolar disorder. Pyruvate enters mitochondria either for oxidation to acetyl-CoA catalyzed by pyruvate dehydrogenase (PDH) or carboxylation to oxaloacetate catalyzed by PC. PC uses bicarbonate as its substrate instead of CO_2_. Carbonic anhydrase (CA) inhibition limits bicarbonate supply to PC. For *de novo* glutamate synthesis in astrocytes, oxaloacetate formed by pyruvate carboxylation condenses with acetyl-CoA and is converted to citrate and subsequently to α-ketoglutarate as part of the tricarboxylic acid (TCA) cycle. α-ketoglutarate can be further converted into glutamate, which was found to be elevated bipolar disorder. Formation of oxaloacetate from pyruvate via PC represents the first committed step of gluconeogenesis which primarily takes place in liver and, to a lesser extent, the cortex of kidneys. For lipogenesis, citrate synthesized via pyruvate carboxylation and subsequent condensation with acetyl-CoA in mitochondria is exported to the cytosol to supply acetyl-CoA for *de novo* fatty acid synthesis. Both fatty acid and triglyceride synthesis take place mainly in liver and adipose tissue.

Pyruvate carboxylase-mediated anaplerosis is at the metabolic crossroad of carbohydrate and lipid metabolism, playing a key role in gluconeogenesis, lipogenesis, and glutamate homeostasis (see Figure 1). In brain, released neurotransmitter glutamate is replenished by the glutamate-glutamine neurotransmitter cycle and *de novo* glutamate synthesis via pyruvate carboxylase-mediated anaplerosis in astrocytes (12–21). In this work we propose that the elevated brain glutamate levels in bipolar disorder observed by ^1^H MRS with very high consistency can be explained by an increase in pyruvate carboxylase-mediated anaplerosis. Evidence supporting increased pyruvate carboxylase-mediated anaplerosis as a common mechanism underlying glutamatergic hyperactivity in bipolar disorder and the positive association between bipolar disorder and obesity is also discussed. The pyruvate carboxylase-mediated anaplerotic pathway may represent future therapeutic targets for bipolar disorder.

## Brain Pyruvate Carboxylation in Bipolar Disorder

### Pyruvate Carboxylase-Mediated Anaplerosis Is Essential for Maintaining Glutamate Homeostasis

Presynaptic release of neurotransmitter glutamate is accompanied by its rapid uptake into astrocytes to maintain an extremely low extracellular glutamate level. The released neuronal glutamate is replenished predominantly by astrocytic glutamine supplied by the glutamate-glutamine neurotransmitter cycle and *de novo* glutamate synthesis (22–24). Abundant evidence shows that neurons lack the anaplerotic enzyme pyruvate carboxylase required for *de novo* synthesis of TCA cycle intermediates (19, 21, 25). Through pyruvate carboxylase-mediated anaplerosis in astrocytes pyruvate and bicarbonate enter the TCA cycle to replenish carbon skeletons lost via glutamine efflux. The subsequently formed TCA cycle intermediate α-ketoglutarate is converted to glutamate by transamination via aspartate aminotransferase or reductive amination via glutamate dehydrogenase. Glutamate can be subsequently converted to glutamine by glutamine synthetase (15), an enzyme exclusively expressed in astrocytes (26). Numerous *in vivo* ^13^C MRS studies have established that the glutamate-glutamine neurotransmitter cycle between astrocytes and neurons is a major metabolic flux in brain (12, 13, 16, 20, 23). In the meanwhile, glutamine efflux from the brain is highly significant (27). Many studies have demonstrated that *de novo* synthesis of glutamate is a significant metabolic pathway essential for maintaining glutamate/glutamine homeostasis in the central nervous system (16, 18).

### Glutamate and Glutamatergic Hyperactivity in Bipolar Disorder

Glutamate is the major excitatory neurotransmitter in the central nervous system. Although the pathophysiology of bipolar disorder is still poorly understood, growing evidence suggests that glutamatergic abnormalities play a key role in the pathogenesis and treatment of bipolar disorder. For example, many rodent studies have demonstrated that mood stabilizers modulate glutamatergic receptors while manipulation of glutamatergic receptors causes significant changes in mood-associated behaviors (28, 29). Post-mortem studies of bipolar disorder have also produced evidence of excitotoxicity in the frontal cortex (30), altered glutamatergic function on both presynaptic and post-synaptic sides, and abnormal excitatory synaptic connections (31, 32). In keeping with the preclinical and post-mortem findings of glutamatergic hyperactivity in bipolar disorder an *in vivo* transcranial magnetic stimulation study has reported impaired cortical inhibition in bipolar disorder (33).

High glutamate + glutamine levels were shown to correlate with cognitive impairment in many brain disorders associated with glutamatergic abnormalities (34). The increased glutamate availability suggests activity-dependent vesicular glutamate release of larger quantal size because vesicle glutamate filling levels are dependent on the concentration of cytoplasmic glutamate to be packaged into synaptic vesicles (35). As excessive glutamate activates ionotropic receptors in extra-synaptic sites and causes neurotoxicity by calcium influx and generation of free radicals including nitric oxide, the sustained elevation of glutamate levels therefore may be a significant part of the pathogenesis of the widespread glutamatergic abnormalities in bipolar disorder (36).

### Elevated Glutamate Levels in Bipolar Disorder Can Be Explained by Increased Pyruvate Carboxylase-Mediated Anaplerosis in Brain

Despite the highly reproducible evidence of elevated brain glutamate levels in bipolar disorder from numerous *in vivo* MRS studies, to the best of our knowledge, a connection between the MRS results and pyruvate carboxylase-mediated anaplerosis has not been made in the literature. However, several drugs used in the treatment of bipolar disorder have important links to pyruvate carboxylase. For example, carbamazepine has long been a therapeutic option for bipolar disorder. It has been used in the treatment of bipolar disorder in both acute mania and maintenance therapy. In rats chronically administered dietary carbamazepine the abundance and activity of biotinylated pyruvate carboxylase were significantly reduced in both liver and brain (37, 38). The potential connection between the efficacy of carbamazepine in bipolar disorder treatment and its effect on pyruvate carboxylase has yet to be investigated.

The important role of the mitochondrial enzyme pyruvate carboxylase in brain function is also well-recognized clinically. Pyruvate carboxylase deficiency, a rare autosomal recessive inborn error of metabolism, is characterized by impairment of lactate metabolism and gluconeogenesis, producing severe lactic acidosis accompanied by compromised psychomotor development and intellectual disability (39). Certain drugs used in the treatment of bipolar disorder improve cerebral metabolism. For example, lithium was demonstrated to enhance oxidative phosphorylation in post-mortem human brain tissue (40) and quetiapine reduced lactate in rapid cycling manic bipolar patients (41).

Despite numerous variations across the studies (e.g., patient selection, disease state, medication history, and ^1^H MRS methodologies) *in vivo* ^1^H MRS studies of bipolar disorder have found elevated glutamate or glutamate + glutamine with surprisingly high consistency (8, 11, 36, 42–45). Consistent, mood phase-independent elevation in glutamate levels in the frontal brain areas was measured in adult bipolar disorder patients by many ^1^H MRS studies (43) while treatment of bipolar disorder patients by lithium and valproate resulted in glutamate + glutamine reduction (45). A meta-analysis (11) of the ^1^H MRS measurement of glutamate + glutamine found elevated glutamate + glutamine levels in bipolar patients when compared with healthy controls with an effect size of 0.72 and a 95% confidence interval of 0.17–1.27 (*p* = 0.01) for the pooled studies that reported glutamate + glutamine in all area of the brain (nine studies with 162 bipolar disorder patients and 165 healthy controls). Analyses of medicated and non-medicated bipolar disorder patients found that the effect size for glutamate level increase in non-medicated patients was much higher (1.91; *p* = 0.03) than in medicated patients (0.31; *p* = 0.03), consistent with that medications decreased brain glutamate. Increased serum α-ketoglutarate and glutamate and increased glutamate in post-mortem brain tissue samples obtained from bipolar disorder individuals have also been reported (46–49). For instance, plasma glutamate levels in patients with bipolar mania (*n* = 20) were significantly higher in both mania phase (46 ± 19 μM, *p* = 0.03) and remission (57 ± 27 μM, *p* = 0.04) than matched controls (36 ± 9 μM, *n* = 20) (46). After correcting for post-mortem changes the level of glutamate at the time of death measured from post-mortem frontal cortex samples (Brodmann area 6) of bipolar disorder patients (15.33 ± 5.72 nmol/mg tissue, *n* = 15) was found to be significantly higher than in the normal control samples (10.68 ± 2.59 nmol/mg tissue, n = 15, *p* = 0.013) (47). In contrast, only a few studies have reported no change in glutamate + glutamine or reduced glutamate + glutamine in brain areas studied (36).

It is well-known in the neurochemical literature that brain relies on pyruvate carboxylase-mediated anaplerosis for *de novo* glutamate synthesis (15–21). Because of the unique role of pyruvate carboxylase in brain glutamate formation the highly consistent findings of elevated glutamate or glutamate + glutamine levels in bipolar disorder observed by *in vivo* ^1^H MRS, serum and post-mortem studies can be readily explained by increased pyruvate carboxylase-mediated anaplerosis in brain of patients with bipolar disorders. This explanation is also supported by the significant comorbidity between bipolar disorder and obesity as described in section Glutamate, Bipolar Disorder, and Comorbid Obesity.

### Elevated Glutamate Levels in Bipolar Disorder Is Consistent With a Chronic Mismatch Between Glucose Utilization and Oxidative Metabolism

A large body of evidence has consistently demonstrated that there is a significant mismatch or uncoupling between glucose utilization and oxidative metabolism in stimulated brain accompanying increased glutamatergic activities (50). Similar mismatches have also been observed in brain after vigorous physical exercise (51). Many functional ^1^H MRS studies have reported transient elevation of glutamate or glutamate + glutamine levels in activated brain tissue in response to stimuli or tasks (52). In preclinical studies, increased glutamatergic activities were found to cause an increase in pyruvate carboxylation, resulting in enlarged glutamate and glutamine pools (17). These results suggest that enhanced glutamatergic activity increases *de novo* synthesis of glutamate from glucose (17). It should be noted that literature evidence for a transient increase in glutamate levels following a functional task or stimulus is not conclusive (52). Recent *in vivo* ^13^C MRS studies of anesthetized rodents found that acute stimulation did not increase pyruvate carboxylase-mediated anaplerotic flux rate in brain (53, 54).

The molar ratio of the arterio-venous difference of oxygen to glucose + ½ lactate is commonly referred to as the oxygen-to-carbohydrate index. The oxygen-to-carbohydrate index is reduced when more glucose and lactate are taken up into the brain than are oxidized to CO_2_. As lactate accumulation can only account for a portion of the large decrease in oxygen-to-carbohydrate index accompanying the mismatch between stimulation of glucose utilization and oxidative metabolism, it has been proposed that increased *de novo* glutamate synthesis via pyruvate carboxylase-mediated anaplerosis contributes to the large decrease in oxygen-to-carbohydrate index when glucose utilization outpaces oxidative metabolism during stimulation of brain activity (17, 51).

^31^P and ^1^H MRS studies have found reduced oxidative phosphorylation and elevated lactate and glutamate + glutamine levels in brain in bipolar disorder, indicating impaired oxidative metabolism (7–10, 36, 42, 43). In contrast, positron emission tomography (PET) studies using [^18^F]fluorodeoxyglucose have reported small or no differences between healthy controls and bipolar disorder patients in glucose utilization rate in the prefrontal cortex or brain as a whole (55–58). There is no consensus in the directionality of the reported differences by the PET studies (55–58). Considering the variations across the PET studies, the lack of consensus in the direction of the changes suggests that the overall abnormalities in cerebral glucose utilization in bipolar disorder are likely very small. The ^31^P and ^1^H MRS and PET results, taken together, indicate that there is a considerable mismatch between oxidative metabolism and glucose utilization in brain in bipolar disorder. Therefore, the elevated glutamate + glutamine levels are consistent with mitochondrial dysfunction and a chronic mismatch between glucose utilization and oxidative metabolism in bipolar disorder accompanied by incomplete carbohydrate oxidation and increased pyruvate carboxylase-mediated anaplerosis.

## Glutamate, Bipolar Disorder, and Comorbid Obesity

### Glutamate Levels and Body Mass Index in Bipolar Disorder

Bipolar disorder and obesity are positively associated (59–62) with cardiovascular disease as the most common cause of death in bipolar disorder patients (63). Bipolar disorder patients are two-thirds more likely to be obese than the age-, race-, and sex-adjusted general population (60). A study of the association between body weight and bipolar illness in drug-naïve patients reported that ~41% of untreated patients with bipolar disorder were overweight or obese (59). Obese bipolar disorder patients also have a more severe mood illness than normal weight patients (61). The underlying causes of the effects of obesity on bipolar disorder are still being investigated (61, 64). Recent neuroimaging studies reported that structural and neurochemical abnormalities in brain characteristic of bipolar disorder were more prominent with higher body mass index (65, 66). In particular, the increase in bilateral hippocampal glutamate + glutamine in patients with first-episode mania measured by ^1^H MRS was found to be more pronounced with higher body mass index (67). In comparison, the correlation between glutamate + glutamine and body mass index in healthy individuals was insignificant (67).

### Obesity Is Associated With Increased Pyruvate Carboxylase-Mediated Anaplerosis

Pyruvate carboxylase plays a crucial role in lipogenesis and gluconeogenesis in mammals. It converts pyruvate and bicarbonate into oxaloacetate for further conversion into citrate which is then exported from mitochondria and cleaved in cytosol to supply precursors for *de novo* fatty acid synthesis [(68); Figure 1]. The activity of pyruvate carboxylase is dramatically increased during adipocyte differentiation. Over expression of pyruvate carboxylase is associated with obesity and type 2 diabetes (69). Of the four gluconeogenic enzymes (phosphoenolpyruvate carboxykinase, fructose-1,6-bisphosphatase, glucose-6-phosphatase, and pyruvate carboxylase) pyruvate carboxylase reaction is the first committed step and likely rate-limiting in gluconeogenesis (70). The pyruvate carboxylase reaction provides oxaloacetate for subsequent conversion into phosphoenolpyruvate by phosphoenolpyruvate carboxykinase and regulates hepatic glucose production. In humans, increased hepatic pyruvate carboxylase expression was closely correlated with plasma glycemia, indicating that hepatic pyruvate carboxylase is a key determinant of gluconeogenesis in liver (71). Animal studies have demonstrated that increased pyruvate carboxylase flux is an important pathway responsible for increased hepatic glucose production in diabetes development (72). Furthermore, selective inhibition of pyruvate carboxylase expression in liver and adipose tissue significantly reduced adiposity, plasma lipid levels and hepatic steatosis (71). A recent *in vivo* ^1^H and ^13^C MRS study of a mouse model of high-fat diet consumption has also found significantly elevated glutamate and glutamate + glutamine levels as well as increased pyruvate carboxylase-mediated anaplerotic flux rate in the hypothalamus of treated animals (73). Taken together, the above evidence demonstrates that increased pyruvate carboxylase-mediated anaplerosis is a metabolic hallmark of obesity.

### Carbonic Anhydrase Inhibition in Bipolar Disorder and Obesity

Catalysis by carbonic anhydrase is necessary to speed up the reversible hydration of CO_2_ for a variety of biological processes. In the central nervous system carbonic anhydrase inhibition enhances inhibitory neurotransmission. Many anticonvulsants are strong carbonic anhydrase inhibitors. Adjunctive acetazolamide, a sulfonamide carbonic anhydrase inhibitor, improved prophylactic efficacy in 44% of the treatment-resistant bipolar disorder patients (74). One of the common adverse effects of acetazolamide is weight loss. Adjunctive topiramate and zonisamide have been used in the treatment of bipolar disorder. They are also strong carbonic anhydrase inhibitors and caused persistent weight loss in obese patients (75–79). Of the three anticonvulsants, the efficacy of topiramate in the treatment of bipolar disorder has been demonstrated by many studies (77). Topiramate also caused substantial weight loss in patients with bipolar disorders in those studies (77).

Inhibition of carbonic anhydrase limits the access of CO_2_-fixing enzymes pyruvate carboxylase and acetyl-CoA carboxylase to bicarbonate and decreases pyruvate carboxylase-mediated anaplerosis in peripheral tissues (Figure 1). It has been demonstrated that carbonic anhydrase activity is required for optimal activity of hepatic pyruvate carboxylase in *de novo* synthesis of both fatty acids and non-saponifiable lipids (80). Carbonic anhydrase inhibitors are known to inhibit *de novo* lipogenesis and gluconeogenesis in liver (81, 82). In cultured adipocytes inhibition of carbonic anhydrase by sulfonamides also significantly decreased lipogenesis (83).

Carbonic anhydrase in brain is predominantly expressed in glial and choroid cells (84–88). The much lesser carbonic anhydrase expression in neurons facilitates rapid removal of CO_2_, which is generated by the highly active neuronal oxidative metabolism, from neurons by free diffusion. This distinct distribution of intracellular carbonic anhydrase in brain leads to the conversion of CO_2_ into bicarbonate primarily in astrocytes, rendering astrocytes as sinks of CO_2_ (89). In cultured astrocytes inhibition of carbonic anhydrase caused a large reduction in pyruvate carboxylase-mediated CO_2_ fixation by limiting the supply of bicarbonate to pyruvate carboxylase, resulting in reduced TCA cycle intermediate levels and reduced glutamate production (90). Since in the central nervous system astrocytes are the predominant site for both CO_2_ hydration catalyzed by carbonic anhydrase and pyruvate carboxylation catalyzed by pyruvate carboxylase (Figure 1), limitation of *de novo* synthesis of glutamate by carbonic anhydrase inhibition may play a significant role in the antiepileptic properties and mood stabilization effects of anticonvulsants that are also carbonic anhydrase inhibitors. Therefore, both mechanistic and clinical studies of carbonic anhydrase inhibition support the proposed connections among bipolar disorder, obesity and pyruvate carboxylase-mediated anaplerosis.

### Pyruvate Carboxylase-Mediated Anaplerosis Is a Potential Therapeutic Target for Bipolar Disorder and Comorbid Obesity

A single pyruvate carboxylase isoform is expressed in humans and found in mitochondria only (91). Pyruvate carboxylase expression is regulated by complex mechanisms and many exogenous and endogenous modulators (80, 91). Many modulators of pyruvate carboxylase pass the blood brain barrier (80) therefore may affect pyruvate carboxylase activities in both peripheral tissues and the brain. Obesity and diabetes are associated with increased pyruvate carboxylase expression in liver and adipose tissue (69). In contrast, insulin inhibits pyruvate carboxylase expression in liver (80). In the central nervous system increased pyruvate supply was found to augment pyruvate carboxylase-mediated anaplerotic flux and glutamate production in astrocytes (15).

Previous studies have shown that pharmacological inhibition of pyruvate carboxylase by phenylacetic acid markedly reduced hepatic gluconeogenesis in rats (92). The effects of pyruvate carboxylase on glucose and lipid metabolism in several rodent models were measured using a specific antisense oligonucleotide to selectively decrease pyruvate carboxylase expression in liver and adipose tissue (71). The specific antisense oligonucleotide approach significantly reduced plasma glucose concentrations and endogenous glucose production. In a high-fat-diet rat model, pyruvate carboxylase antisense oligonucleotide reduced adiposity, plasma lipid levels, and hepatic steatosis (71). It has been suggested that pyruvate carboxylase is a potential therapeutic target for several diseases associated with obesity (71, 92). As the experimental findings discussed here indicate that elevated pyruvate carboxylation may be a significant part of the pathogenesis of glutamatergic hyperactivity and comorbid obesity in bipolar disorder, designing inhibitors of pyruvate carboxylase to pharmacologically modulate pyruvate carboxylase-mediated anaplerosis may be a useful new treatment strategy for bipolar disorder and comorbid obesity.

## Conclusions

Increased pyruvate carboxylase-mediated anaplerosis can readily explain the elevated glutamate or glutamate + glutamine levels in brain in bipolar disorder observed by *in vivo* ^1^H MRS. Multiple independent lines of evidence suggest that increased pyruvate carboxylase-mediated anaplerosis is a common mechanism underlying glutamatergic hyperactivity and the significant positive association between bipolar disorder and obesity. As the increased prevalence of obesity in bipolar disorder is associated with illness severity and poor treatment outcomes development of preventive and treatment strategies targeting pyruvate carboxylase-mediated anaplerosis may be warranted.

## Author Contributions

JS performed literature analysis and proposed the hypothesis. JT performed literature search. JS and JT wrote the paper. Both authors reviewed the manuscript and agreed on its final version.

## Conflict of Interest

The authors declare that the research was conducted in the absence of any commercial or financial relationships that could be construed as a potential conflict of interest.

## References

[1] Cataldo AM McPhie DL Lange NT Punzell S Elmiligy S Ye NZ. Abnormalities in mitochondrial structure in cells from patients with bipolar disorder. Am J Pathol. (2010) 177:575–85. 10.2353/ajpath.2010.081068 20566748 PMC2913344

[2] Konradi C Eaton M MacDonald ML Walsh J Benes FM Stephan Heckers S. Molecular evidence for mitochondrial dysfunction in bipolar disorder. Arch Gen Psychiatry. (2004) 61:301–8. 10.1001/archpsyc.61.3.300 14993118

[3] Mertens J Wang QW Kim Y Yu DX Pham S Yang B. Differential responses to lithium in hyperexcitable neurons from patients with bipolar disorder. Nature. (2015) 527:95–9. 10.1038/nature15526 26524527 PMC4742055

[4] Regenold WT Phatak P Marano CM Sassan A Conley RR Kling MA. Elevated cerebrospinal fluid lactate concentrations in patients with bipolar disorder and schizophrenia: implications for the mitochondrial dysfunction hypothesis. Biol Psychiatry. (2009) 65:489–94. 10.1016/j.biopsych.2008.11.010 19103439 PMC3752997

[5] Yoshimi N Futamura T Bergen SE Iwayama Y Ishima T Sellgren C. Cerebrospinal fluid metabolomics identifies a key role of isocitrate dehydrogenase in bipolar disorder: evidence in support of mitochondrial dysfunction hypothesis. Mol Psychiatry. (2016) 21:1504–10. 10.1038/mp.2015.217 26782057 PMC5078854

[6] Kato T Kato N. Mitochondrial dysfunction in bipolar disorder. Bipolar Disord. (2000) 2:180–90. 10.1034/j.1399-5618.2000.020305.x 11256685

[7] Dager SR Friedman SD Parow A Demopulos C Stoll AL Lyoo IK. Brain metabolic alterations in medication-free patients with bipolar disorder. Arch Gen Psychiatry. (2004) 61:450–8. 10.1001/archpsyc.61.5.450 15123489

[8] Stork C Renshaw PF. Mitochondrial dysfunction in bipolar disorder: evidence from magnetic resonance spectroscopy research. Mol Psychiatry. (2005) 10:900–19. 10.1038/sj.mp.4001711 16027739

[9] Frey BN Stanley JA Nery FG Serap Monkul E Nicoletti MA Chen HH. Abnormal cellular energy and phospholipid metabolism in the left dorsolateral prefrontal cortex of medication-free individuals with bipolar disorder: an *in vivo* 1H MRS study. Bipolar Disord. (2007) 9:119–27. 10.1111/j.1399-5618.2007.00454.x 17543030

[10] Chu WJ DelBello MP Jarvis KB Norris MM Kim M-J Weber W. Magnetic resonance spectroscopy imaging of lactate in patients with bipolar disorder. Psychiatry Res. (2013) 213:230–4. 10.1016/j.pscychresns.2013.03.004 23810640

[11] Gigante AD Bond DJ Lafer B Lam RW Young LT Yatham LN. Brain glutamate levels measured by magnetic resonance spectroscopy in patients with bipolar disorder: a meta-analysis. Bipolar Disord. (2012) 14:478–87. 10.1111/j.1399-5618.2012.01033.x 22834460

[12] Sibson NR Dhankhar A Mason GF Behar KL Rothman DL Shulman RG. *In vivo* ^13^C NMR measurements of cerebral glutamine synthesis as evidence for glutamate-glutamine cycling. Proc Natl Acad Sci USA. (1997) 94:2699–704. 10.1073/pnas.94.6.2699 9122259 PMC20152

[13] Rothman DL de Graaf RA Hyder F Mason GF Behar KL De Feyter HM. *In vivo* ^13^C and ^1^H-[^13^C] MRS studies of neuroenergetics and neurotransmitter cycling, applications to neurological and psychiatric disease and brain cancer. NMR Biomed. (2019) 32:e4172. 10.1002/nbm.4172 31478594

[14] Pardo B Contreras L Satrustegui J. *De novo* synthesis of glial glutamate and glutamine in young mice requires aspartate provided by the neuronal mitochondrial aspartate-glutamate carrier aralar/AGC1. Front Endocrinol. (2013) 4:149. 10.3389/fendo.2013.00149 24133485 PMC3796713

[15] Gamberino WC Berkich DA Lynch CJ Xu B LaNoue KF. Role of pyruvate carboxylase in facilitation of synthesis of glutamate and glutamine in cultured astrocytes. J Neurochem. (1997) 69:2312–25. 10.1046/j.1471-4159.1997.69062312.x 9375662

[16] Sibson NR Mason GF Shen J Cline GW Herskovits AZ Wall JE. *In vivo* (13)C NMR measurement of neurotransmitter glutamate cycling, anaplerosis and TCA cycle flux in rat brain during [2-13C]glucose infusion. J Neurochem. (2001) 76:975–89. 10.1046/j.1471-4159.2001.00074.x 11181817

[17] Hertz L. Intercellular metabolic compartmentation in the brain: past, present and future. Neurochem Int. (2004) 45:285–96. 10.1016/j.neuint.2003.08.016 15145544

[18] Lapidot A Gopher A. Cerebral metabolic compartmentation. Estimation of glucose flux via pyruvate carboxylase/pyruvate dehydrogenase by ^13^C NMR isotopomer analysis of D-[U-^13^C]glucose metabolites. J Biol Chem. (1994) 269:27198–208. 10.1016/S0021-9258(18)46969-4 7961629

[19] Shank RP Bennett GS Freytag SO Campbell GL. Pyruvate carboxylase: an astrocyte-specific enzyme implicated in the replenishment of amino acid neurotransmitter pools. Brain Res. (1985) 329:364–7. 10.1016/0006-8993(85)90552-9 3884090

[20] Shen J. Modeling the glutamate-glutamine neurotransmitter cycle. Front Neuroenergetics. (2013) 5:1. 10.3389/fnene.2013.00001 23372548 PMC3556573

[21] Waagepetersen HS Qu H Schousboe A Sonnewald U. Elucidation of the quantitative significance of pyruvate carboxylation in cultured cerebellar neurons and astrocytes. J Neurosci Res. (2001) 66:763–70. 10.1002/jnr.10061 11746400

[22] Hertz L. Functional interactions between neurons and astrocytes I. Turnover and metabolism of putative amino acid transmitters. Prog Neurobiol. (1979) 13:277–323. 10.1016/0301-0082(79)90018-2 42117

[23] Rothman DL Sibson NR Hyder F Shen J Behar KL Shulman RG. *In vivo* nuclear magnetic resonance spectroscopy studies of the relationship between the glutamate-glutamine neurotransmitter cycle and functional neuroenergetics. Philos Trans R Soc Lond B. (1999) 354:1165–77. 10.1098/rstb.1999.0472 10466144 PMC1692640

[24] Shen J. ^13^C MRS studies of alterations in glutamate neurotransmission. Biol Psychiatry. (2006) 59:883–7. 10.1016/j.biopsych.2005.07.042 16199016

[25] Cesar M Hamprecht B. Immunocytochemical examination of neural rat and mouse primary cultures using monoclonal antibodies raised against pyruvate carboxylase. J Neurochem. (1995) 64:2312–8. 10.1046/j.1471-4159.1995.64052312.x 7722517

[26] Martinez-Hernandez A Bell KP Norenberg MD. Glutamine synthetase: glial localization in brain. Science. (1977) 195:1356–8. 10.1126/science.14400 14400

[27] Grill V Bjorkman O Gutniak M Lindqvist M. Brain uptake and release of amino acids in nondiabetic and insulin-dependent diabetic subjects: important role of glutamine release for nitrogen balance. Metabolism. (1992) 41:28–32. 10.1016/0026-0495(92)90186-E 1538641

[28] Lapidus KA Soleimani L Murrough JW. Novel glutamatergic drugs for the treatment of mood disorders. Neuropsychiatr Dis Treat. (2013) 9:1101–12. 10.2147/NDT.S36689 23976856 PMC3747027

[29] Du J Wei Y Liu L Wang Y Khairova R Blumenthal R. A kinesin signaling complex mediates the ability of GSK-3beta to affect mood-associated behaviors. Proc Natl Acad Sci USA. (2010) 107:11573–8. 10.1073/pnas.0913138107 20534517 PMC2895136

[30] Rao JS Harry GJ Rapoport SI Kim HW. Increased excitotoxicity and neuroinflammatory markers in postmortem frontal cortex from bipolar disorder patients. Mol Psychiatry. (2009) 15:384–92. 10.1038/mp.2009.47 19488045 PMC2844920

[31] Eastwood SL Harrison PJ. Markers of glutamate synaptic transmission and plasticity are increased in the anterior cingulate cortex in bipolar disorder. Biol Psychiatry. (2010) 67:1010–6. 10.1016/j.biopsych.2009.12.004 20079890 PMC2868790

[32] Jun C Choi Y Lim SM Bae S Hong YS Kim JE. Disturbance of the glutamatergic system in mood disorders. Exp. Neurobiol. (2014) 23:28–35. 10.5607/en.2014.23.1.28 24737937 PMC3984954

[33] Levinson AJ Young LT Fitzgerald PB Daskalakis ZJ. Cortical inhibitory dysfunction in bipolar disorder: a study using transcranial magnetic stimulation. J Clin Psychopharmacol. (2007) 27:493–7. 10.1097/jcp.0b013e31814ce524 17873683

[34] Fayed N Andres E Viguera L Modrego PJ Garcia-Campayo J. Higher glutamate+glutamine and reduction of N-acetylaspartate in posterior cingulate according to age range in patients with cognitive impairment and/or pain. Acad Radiol. (2014) 21:1211–7. 10.1016/j.acra.2014.04.009 24981958

[35] Pietrancosta N Djibo M Daumas S El Mestikawy S Erickson JD. Molecular, structural, functional, and pharmacological sites for vesicular glutamate transporter regulation. Mol. Neurobiol. (2020) 30:1–25. 10.1007/s12035-020-01912-7 32474835 PMC7261050

[36] Yüksel C Öngür D. Magnetic resonance spectroscopy studies of glutamate-related abnormalities in mood disorders. Biol Psychiatry. (2010) 68:785–94. 10.1016/j.biopsych.2010.06.016 20728076 PMC2955841

[37] Rathman SC Eisenschenk S McMahon RJ. The abundance and function of biotin-dependent enzymes are reduced in rats chronically administered carbamazepine. J Nutr. (2002) 132:3405–10. 10.1093/jn/132.11.3405 12421859

[38] Rathman SC Gregory JF McMahon RJ. Pharmacological biotin supplementation maintains biotin status and function in rats administered dietary carbamazepine. J Nutr. (2003) 133:2857–62. 10.1093/jn/133.9.2857 12949377

[39] Marin-Valencia I Roe CR Pascual JM. Pyruvate carboxylase deficiency: mechanisms, mimics and anaplerosis. Mol Genet Metab. (2010) 101:9–17. 10.1016/j.ymgme.2010.05.004 20598931

[40] Maurer IC Schippel P Volz HP. Lithium-induced enhancement of mitochondrial oxidative phosphorylation in human brain tissue. Bipolar Disord. (2009) 11:515–22. 10.1111/j.1399-5618.2009.00729.x 19624390

[41] Kim DJ Lyoo IK Yoon SJ Choi T Lee B Kim JE. Clinical response of quetiapine in rapid cycling manic bipolar patients and lactate level changes in proton magnetic resonance spectroscopy. Prog Neuropsychopharmacol Biol Psychiatry. (2007) 31:1182–8. 10.1016/j.pnpbp.2007.04.009 17532107 PMC2731791

[42] Ehrlich A Schubert F Pehrs C Gallinat J. Alterations of cerebral glutamate in the euthymic state of patients with bipolar disorder. Psychiatry Res. (2015) 233:73–80. 10.1016/j.pscychresns.2015.05.010 26050195

[43] Chitty KM Lagopoulos J Lee RS Hickie IB Hermens DF. A systematic review and meta-analysis of proton magnetic resonance spectroscopy and mismatch negativity in bipolar disorder. Eur Neuropsychopharmacol. (2013) 23:1348–63. 10.1016/j.euroneuro.2013.07.007 23968965

[44] Kim Y Santos R Gage FH Marchetto MC. Molecular mechanisms of bipolar disorder: progress made and future challenges. Front Cell Neurosci. (2017) 11:30. 10.3389/fncel.2017.00030 28261061 PMC5306135

[45] Friedman S Dager S Parow A Hirashima F Demopulos C Stoll AL. Lithium and valproic acid treatment effects on brain chemistry in bipolar disorder. Biol Psychiatry. (2004) 56:340–48. 10.1016/j.biopsych.2004.06.012 15336516

[46] Hoekstra R Fekkes D Loonen A Pepplinkhuizen L Tuinier S Verhoeven W. Bipolar mania and plasma amino acids: increased levels of glycine. Eur Neuropsychopharmacol. (2006) 16:71–7. 10.1016/j.euroneuro.2005.06.003 16023835

[47] Hashimoto K Sawa A Iyo M. Increased levels of glutamate in brains from patients with mood disorders. Biol Psychiatry. (2007) 62:1310–6. 10.1016/j.biopsych.2007.03.017 17574216

[48] Lan MJ McLoughlin GA Griffin JL Tsang TM Huang JTJ Yuan P. Metabonomic analysis identifies molecular changes associated with the pathophysiology and drug treatment of bipolar disorder. Mol. Psychiatry. (2009) 14:269–79. 10.1038/sj.mp.4002130 18256615

[49] Yoshimi N Futamura T Kakumoto K Salehi AM Sellgren CM HolménLarsson J. Blood metabolomics analysis identifies abnormalities in the citric acid cycle, urea cycle and amino acid metabolism in bipolar disorder. BBA Clin. (2016) 5:151–8. 10.1016/j.bbacli.2016.03.008 27114925 PMC4832124

[50] Fox PT Raichle ME. Focal physiological uncoupling of cerebral blood flow and oxidative metabolism during somatosensory stimulation in human subjects. Proc Natl Acad Sci USA. (1986) 83:1140–4. 10.1073/pnas.83.4.1140 3485282 PMC323027

[51] Maddock RJ Casazza GA Buonocore MH Tanase C. Vigorous exercise increases brain lactate and Glx (glutamate + glutamine): a dynamic 1H-MRS study. NeuroImage. (2011) 57:1324–30. 10.1016/j.neuroimage.2011.05.048 21640838

[52] Duncan NW Wiebking C Northoff G. Associations of regional GABA and glutamate with intrinsic and extrinsic neural activity in humans—a review of multimodal imaging studies. Neurosci Biobehav Rev. (2014) 47:36–52. 10.1016/j.neubiorev.2014.07.016 25066091

[53] Sonnay S Duarte JMN Just N Gruetter R. Compartmentalised energy metabolism supporting glutamatergic neurotransmission in response to increased activity in the rat cerebral cortex: A ^13^C MRS study *in vivo* at 14.1 T. J Cereb Blood Flow Metab. (2016) 6:928–40. 10.1177/0271678X16629482 PMC4853840 26823472

[54] Sonnay S Poirot J Just N Clerc AC Gruetter R. Astrocytic and neuronal oxidative metabolism are coupled to the rate of glutamate–glutamine cycle in the tree shrew visual cortex. Glia. (2018) 66:477–91. 10.1002/glia.23259 29120073

[55] Ketter TA George MS Kimbrell TA Benson BE Post RM. Functional brain imaging, limbic function, and affective disorders. Neuroscientist. (1996) 2:55–65. 10.1177/107385849600200113

[56] Ketter TA Kimbrell TA George MS Dunn RT Speer AM Benson BE. Effects of mood and subtype on cerebral glucose metabolism in treatment-resistant bipolar disorder. Biol Psychiatry. (2001) 49:97–109. 10.1016/S0006-3223(00)00975-6 11164756

[57] Soares JC Mann JJ. The functional neuroanatomy of mood disorders. J Psychiatr Res. (1997) 31:393–432. 10.1016/S0022-3956(97)00016-2 9352470

[58] Baxter LR Jr Schwartz JM Phelps ME Mazziotta JC Guze BH Selin CE. Ruction of prefrontal cortex glucose metabolism common to three types of depression. Arch Gen Psychiatry. (1989) 46:243–50. 10.1001/archpsyc.1989.01810030049007 2784046

[59] Maina G Salvi V Vitalucci A D'Ambrosio V Bogetto F. Prevalence and correlates of overweight in drug-naive patients with bipolar disorder. J Affect Disord. (2008) 110:149–55. 10.1016/j.jad.2007.12.233 18234351

[60] Goldstein BI Liu SM Zivkovic N Schaffer A Chien LC Blanco C. The burden of obesity among adults with bipolar disorder in the United States. Bipolar Disord. (2011) 13:387–95. 10.1111/j.1399-5618.2011.00932.x 21843278 PMC3157038

[61] McElroy SL Keck PE Jr. Obesity in bipolar disorder: an overview. Curr Psychiatry Rep. (2012) 14:650–8. 10.1007/s11920-012-0313-8 22903246

[62] Zhao Z Okusaga OO Quevedo J Soares JC Teixeira AL. The potential association between obesity and bipolar disorder: a meta-analysis. J Affect Disord. (2016) 202:120–3. 10.1016/j.jad.2016.05.059 27262632

[63] Weiner M Warren L Fiedorowicz JG. Cardiovascular morbidity and mortality in bipolar disorder. Ann Clin Psychiatry. (2011) 23:40–7. 21318195 PMC3190964

[64] Taylor VH McIntyre RS Remington G Levitan RD Stonehocker B Sharma AM. Beyond pharmacotherapy: understanding the links between obesity and chronic mental illness. Can J Psychiatry. (2012) 57:5–12. 10.1177/070674371205700103 22296962

[65] Kuswanto CN Sum MY Yang GL Nowinski WL McIntyre RS Sim K. Increased body mass index makes an impact on brain white-matter integrity in adults with remitted first-episode mania. Psychol Med. (2014) 44:533–41. 10.1017/S0033291713000858 23731622

[66] Bond DJ da Silveira LE MacMillan EL Torres IJ Lang DJ Su W. Diagnosis and body mass index effects on hippocampal volumes and neurochemistry in bipolar disorder. Transl Psychiatry. (2017) 7:e1071. 10.1038/tp.2017.42 28350397 PMC5404613

[67] Bond DJ da Silveira LE MacMillan EL Torres IJ Lang DJ Su W. Relationship between body mass index and hippocampal glutamate/glutamine in bipolar disorder. Br J Psychiatry. (2016) 208:146–52. 10.1192/bjp.bp.115.163360 26585092

[68] Mackall JC Lane MD. Role of pyruvate carboxylase in fatty acid synthesis: alterations during preadipocyte differentiation. Biochem Biophys Res Commun. (1977) 79:720–5. 10.1016/0006-291X(77)91171-8 597301

[69] Lynch CJ McCall KM Billingsley ML Bohlen LM Hreniuk SP Martin LF. Pyruvate carboxylase in genetic obesity. Am J Physiol. (1992) 262:E608–18. 10.1152/ajpendo.1992.262.5.E608 1375435

[70] Attwood PA Keech DB. Pyruvate carboxylase. Curr Top Cell Regul. (1984) 23:1–55. 10.1016/B978-0-12-152823-2.50005-2 6373162

[71] Kumashiro N Beddow SA Vatner DF Majumdar SK Cantley JL Guebre-Egziabher F. Targeting pyruvate carboxylase reduces gluconeogenesis and adiposity and improves insulin resistance. Diabetes. (2013) 62:2183–94. 10.2337/db12-1311 23423574 PMC3712050

[72] Lee P Leong W Tan T Lim M Han W Radda GK. *In vivo* hyperpolarized carbon-13 magnetic resonance spectroscopy reveals increased pyruvate carboxylase flux in an insulin-resistant mouse model. Hepatol. (2013) 57:515–24. 10.1002/hep.26028 22911492

[73] Lizarbre B Cherix A Duarte JMN Cardinaux JR Gruetter R. High-fat diet consumption alters energy metabolism in the mouse hypothalamus. Intl J Obesity. (2019) 43:1295–304. 10.1038/s41366-018-0224-9 30301962

[74] Hayes SG. Acetazolamide in bipolar affective disorders. Ann Clin Psychiatry. (1994) 6:91–8. 10.3109/10401239409148987 7804393

[75] McElroy SL Suppes T Keck PE Jr. Frye MA Denicoff KD Altshuler LL. Open-label adjunctive topiramate in the treatment of bipolar disorders. Biol Psychiatry. (2000) 47:1025–33. 10.1016/S0006-3223(99)00316-9 10862801

[76] Gadde KM Kopping MF Wagner HR Yonish GM Allison DB Bray GA. Zonisamide for weight reduction in obese adults a 1-year randomized controlled trial. Arch Intern Med. (2012) 172:1557–64. 10.1001/2013.jamainternmed.99 23147455 PMC3753218

[77] Grootens KP Meijer A Hartong EG Doornbos B Bakker PR Al Hadithy A. Weight changes associated with antiepileptic mood stabilizers in the treatment of bipolar disorder. Eur J Clin Pharmacol. (2018) 74:1485–9. 10.1007/s00228-018-2517-2 30083876

[78] Gupta S Masand PS Frank BL Lockwood KL Keller PL. Topiramate in bipolar and schizoaffective disorders: weight loss and efficacy, Prim Care Companion J. Clin. Psychiatry. (2000) 2:96–100. 10.4088/PCC.v02n0304 15014655 PMC181115

[79] Dodgson SJ Shank RP Maryanoff BE. Topiramate as an inhibitor of carbonic anhydrase isoenzymes. Epilepsia. (2000) 41(Suppl. 1):S35–9. 10.1111/j.1528-1157.2000.tb02169.x 10768298

[80] Jitrapakdee S St Maurice M Rayment I Cleland Wallace WW Attwood JC. Structure, mechanism and regulation of pyruvate carboxylase. Biochem J. (2008) 413:369–87. 10.1042/BJ20080709 18613815 PMC2859305

[81] Dodgson SJ Forster RE. Inhibition of CA V decreases glucose synthesis from pyruvate. Arch Biochem Biophys. (1986) 251:198–204. 10.1016/0003-9861(86)90066-4 3098176

[82] Lynch CJ Fox H Hazcn SA Stanley BA Dodgson SJ LaNouc KF. The role of hepatic carbonic anhydrase in de novo lipogenesis. Biochem J. (1995) 310:197–202. 10.1042/bj3100197 7646445 PMC1135873

[83] Hazen SA Waheed A Sly WS LaNoue KF Lynch CJ. Differentiation-dependent expression of CA V and the role of carbonic anhydrase isozymes in pyruvate carboxylation in adipocytes. FASEB J. (1996) 10:481–90. 10.1096/fasebj.10.4.8647347 8647347

[84] Giacobini E. A cytochemical study of the localization of carbonic anhydrase in the nervous system. J Neurochem. (1962) 9:169–77. 10.1111/j.1471-4159.1962.tb11859.x 13898264

[85] Sapirstein VS Strocchi P Gilbert JM. Properties and function of brain carbonic anhydrase. Ann NY Acad Sci. (1984) 429:481–93. 10.1111/j.1749-6632.1984.tb12375.x 6430185

[86] Kumpulainen T. Immunohistochemical localization of human carbonic anhydrase isozymes. Ann NY Acad Sci. (1984) 429:359–67. 10.1111/j.1749-6632.1984.tb12360.x 6430174

[87] Cammer W. Immunostaining of carbamoylphosphate synthase II and fatty acid synthase in glial cells in rat, mouse, and hamster brains suggests roles for carbonic anhydrase in biosynthetic processes. Neurosci Lett. (1991) 129:247–50. 10.1016/0304-3940(91)90472-6 1684028

[88] Agnati LF Tinner B Staines WA Vaananen K Fuxe K. On the cellular localization and distribution of carbonic anhydrase II immunoreactivity in the rat brain. Brain Res. (1995) 676:10–24. 10.1016/0006-8993(95)00026-M 7796160

[89] Deitmer JW. Strategies for metabolic exchange between glial cells and neurons. Respir Physiol. (2001) 129:71–81. 10.1016/S0034-5687(01)00283-3 11738647

[90] Hazen SA Waheed A Sly WS LaNoue KF Lynch CJ. Effect of carbonic anhydrase inhibition and acetoacetate on anaplerotic pyruvate carboxylase activity in cultured rat astrocytes. Dev Neurosci. (1997) 19:162–71. 10.1159/000111202 9097031

[91] Gray LR Tompkins SC Taylor EB. Regulation of pyruvate metabolism and human disease. Cell Mol Life Sci. (2014) 71:2577–604. 10.1007/s00018-013-1539-2 24363178 PMC4059968

[92] Bahl JJ Matsuda M DeFronzo RA Bressler R. *In vitro* and *in vivo* suppression of gluconeogenesis by inhibition of pyruvate carboxylase. Biochem Pharmacol. (1997) 53:67–74. 10.1016/S0006-2952(96)00660-0 8960065

